# He’s Come!!

**Published:** 1869-04

**Authors:** A. T. Stewart


### HE 'S COME!!

The coming man is here. He is making his mark for all time sooner than was expected, and with a bigger heart and a larger, grander, nobler purpose than the great public had looked for. He worked hard, was up late and early, and always busy. He made no noise about it. He was none of the "fussy kind;" no blusterer. No man ever saw him in a hurry; always quiet; always composed, always sedate. A stranger would never have singled him out of any crowd as a king of his kind. He drinks no wine, uses no tobacco, drives no four-in-hand up the avenue, wears no diamonds, sports no gold-headed cane; the newly rich delight in vanities like these. His mind was on business; day and night, summer and winter, from one decade to another, his whole soul has been intent on business. What is it for? What is he going to do with his accumulating millions? He has no children, no near relations. Why don 't he retire and leave the field for others who are striving ever so hard for bread, but whom he overshadows and "cuts out?" This is the way the people talk about him, and then they go on to soliloquise: Why don 't he build houses at a cheap rent for poor men with families to support, clerks, mechanics and others, and thus do some good with his money? Some have gone so far, presuming on their acquaintance with him, as to suggest that this would be a good plan, and that would be a good way to immortalize himself, and do a great deal of good besides. But he did not seem to want to be immortalized, and went on accumulating other millions. Yet all this while he appears to have been busy clearing away the rubbish, laying his own plans, and perfecting his own line of operations. He is one of the few men who does not begin until he is ready, and then goes ahead in good earnest. He is long-headed, looks far into the future, studies cause and effect, and takes time by the forelock. He could have given many thousands of poor men with large families a good sized farm apiece ; he might have helped thousands of struggling widows, or aided multitudes of young men of promise and talent in securing an education ; but he saw that others were engaged in charities like these. None of them seemed to him to go to the root of the matter ; they neither eradicated nor prevented one of the thousand social evils which afflict the body politic ; they were only temporary alleviants. He seemed to want to help the most helpless, to be a friend to the most unfriended, to give aid to those who needed it most, and at a time so early that it would be efficient in preventing destitution, sickness and wasting, wearing poverty. He seems to have found out that the more you help the passively helpless, the more they expect to be helped, and in a short time claim such help as a right, and are wonderfully offended if their increasing demands are not met. At one fell swoop he cut off from his charities one half of the whole human family. He said, "let the men help themselves." At another stroke of his pen he erased from his benefactions more than one half of the whole remainder—one half the women : "let those who are married look to their husbands ;" one half the remainder of the daughters of the land had parents or brothers who could take care of them ; half of the comparatively small remainder he rejected as being unworthy of his sympathies, to wit : those who were not willing to work for a living. But to unbefriended and destitute young women, who were willing to work for a living, he has decided to give millions of money, and in a way not to encourage idleness, nor a whit to abate their industries ; but to every young woman who has an ambition to make and save money, he says : "I will furnish you a nice, warm, well lighted, airy room, suitably furnished, in a splendid marble house, with good and healthful board, for two dollars and a half a week; all that you can make beyond that is your own." Does not the reader see at a glance the wisdom, the beneficence and the gallantry of a plan of this kind? He affords industrious young women an opportunity of making and laying up money for themselves; he does nothing towards encouraging them in idleness; nothing to lower their self-respect. If he gave them their board and lodging they could not help but consider themselves objects of charity, and there would be more or less of a feeling of self-degradation, or that they were "beholden" to another. It is difficult to compute the amount of actual good this plan will accomplish; to compute the amount of discouragement and premature sickness, and the lowest of all depravities which will be prevented every year, and to be continued for ages to come. Such is the general outline of the plan now in process of execution at the northwest corner of Fourth Avenue and Thirty-second street, in New York city, to be carried out at the cost of several millions of dollars by that prince of merchants, and, at this writing, and since the commencement of this article, appointed by the President of the United States to the highest and most responsible position next to his own. We mean the

HON. A. T. STEWART,

Secretary of the Treasury of the United States, having been unanimously confirmed by the Senate of the nation, and to the satisfaction of the whole people.

